# Supplementary material for: OpenMM 7: Rapid development of high performance algorithms for molecular dynamics
Source: PLoS Comput Biol. 2017 Jul 26;13(7):e1005659. doi: 10.1371/journal.pcbi.1005659 (PMC5549999; doi:10.1371/journal.pcbi.1005659)
Supplement: S1 Source Code — (ZIP) [file pcbi.1005659.s002.zip › OpenMM-7.0.1-Source/docs/api-python/generated/simtk.openmm.openmm.CustomHbondForce.html]

CustomHbondForce — OpenMM 7.0.1.dev-5e86c4f documentation


# CustomHbondForce¶

*class* `simtk.openmm.openmm.``CustomHbondForce`(*\*args*)¶
:   This class supports a wide variety of energy functions used to represent hydrogen bonding. It computes interactions between “donor” particle groups and “acceptor” particle groups, where each group may include up to three particles. Typically a donor group consists of a hydrogen atom and the atoms it is bonded to, and an acceptor group consists of a negatively charged atom and the atoms it is bonded to.

    We refer to the particles in a donor group as d1, d2 and d3, and the particles in an acceptor group as a1, a2, and a3. For each donor and each acceptor, CustomHbondForce evaluates a user supplied algebraic expression to determine the interaction energy. The expression may depend on arbitrary distances, angles, and dihedral angles defined by any of the six particles involved. The function distance(p1, p2) is the distance between the particles p1 and p2 (where “p1” and “p2” should be replaced by the names of the actual particles to calculate the distance between), angle(p1, p2, p3) is the angle formed by the three specified particles, and dihedral(p1, p2, p3, p4) is the dihedral angle formed by the four specified particles.

    The expression also may involve tabulated functions, and may depend on arbitrary global, per-donor, and per-acceptor parameters. It also optionally supports periodic boundary conditions and cutoffs for long range interactions.

    To use this class, create a CustomHbondForce object, passing an algebraic expression to the constructor that defines the interaction energy between each donor and acceptor. Then call addPerDonorParameter() to define per-donor parameters, addPerAcceptorParameter() to define per-acceptor parameters, and addGlobalParameter() to define global parameters. The values of per-donor and per-acceptor parameters are specified as part of the system definition, while values of global parameters may be modified during a simulation by calling Context::setParameter().

    Next, call addDonor() and addAcceptor() to define donors and acceptors and specify their parameter values. After a donor or acceptor has been added, you can modify its parameters by calling setDonorParameters() or setAcceptorParameters(). This will have no effect on Contexts that already exist unless you call updateParametersInContext().

    CustomHbondForce also lets you specify “exclusions”, particular combinations of donors and acceptors whose interactions should be omitted from force and energy calculations. This is most often used for particles that are bonded to each other.

    As an example, the following code creates a CustomHbondForce that implements a simple harmonic potential to keep the distance between a1 and d1, and the angle formed by a1-d1-d2, near ideal values:

    ```
    CustomHbondForce* force = new CustomHbondForce("k*(distance(a1,d1)-r0)^2*(angle(a1,d1,d2)-theta0)^2");
    ```

    This force depends on three parameters: k, r0, and theta0. The following code defines these as per-donor parameters:

    ```
    force->addPerDonorParameter("k");
    force->addPerDonorParameter("r0");
    force->addPerDonorParameter("theta0");
    ```

    Expressions may involve the operators + (add), - (subtract), \* (multiply), / (divide), and ^ (power), and the following functions: sqrt, exp, log, sin, cos, sec, csc, tan, cot, asin, acos, atan, sinh, cosh, tanh, erf, erfc, min, max, abs, floor, ceil, step, delta, select. All trigonometric functions are defined in radians, and log is the natural logarithm. step(x) = 0 if x is less than 0, 1 otherwise. delta(x) = 1 if x is 0, 0 otherwise. select(x,y,z) = z if x = 0, y otherwise.

    In addition, you can call addTabulatedFunction() to define a new function based on tabulated values. You specify the function by creating a TabulatedFunction object. That function can then appear in the expression.

    `__init__`(*self*, *energy*) → CustomHbondForce¶
    :   \_\_init\_\_(self, other) -> CustomHbondForce

        Create a CustomHbondForce.

        |  |  |
        | --- | --- |
        | Parameters: | **energy** (*string*) – an algebraic expression giving the interaction energy between a donor and an acceptor as a function of inter-particle distances, angles, and dihedrals, as well as any global, per-donor, and per-acceptor parameters |

    Methods

    |  |  |
    | --- | --- |
    | `__init__`((self, energy) -> CustomHbondForce) | \_\_init\_\_(self, other) -> CustomHbondForce |
    | `addAcceptor`((self, a1, a2, a3, ...) | addAcceptor(self, a1, a2, a3) -> int |
    | `addDonor`((self, d1, d2, d3, parameters) -> int) | addDonor(self, d1, d2, d3) -> int |
    | `addExclusion`((self, donor, acceptor) -> int) | Add a donor-acceptor pair to the list of interactions that should be excluded. |
    | `addFunction`((self, name, values, min, ...) | Add a tabulated function that may appear in the energy expression. |
    | `addGlobalParameter`((self, name, ...) | Add a new global parameter that the interaction may depend on. |
    | `addPerAcceptorParameter`((self, name) -> int) | Add a new per-acceptor parameter that the interaction may depend on. |
    | `addPerDonorParameter`((self, name) -> int) | Add a new per-donor parameter that the interaction may depend on. |
    | `addTabulatedFunction`((self, name, ...) | Add a tabulated function that may appear in the energy expression. |
    | `getAcceptorParameters`(self, index) | Get the properties of an acceptor group. |
    | `getCutoffDistance`((self) -> double) | Get the cutoff distance (in nm) being used. |
    | `getDonorParameters`(self, index) | Get the properties of a donor group. |
    | `getEnergyFunction`((self) -> std::string const &) | Get the algebraic expression that gives the interaction energy between a donor and an acceptor |
    | `getExclusionParticles`(self, index) | Get the donor and acceptor in a pair whose interaction should be excluded. |
    | `getForceGroup`((self) -> int) | Get the force group this Force belongs to. |
    | `getFunctionParameters`(self, index) | Get the parameters for a tabulated function that may appear in the energy expression. |
    | `getGlobalParameterDefaultValue`((self, ...) | Get the default value of a global parameter. |
    | `getGlobalParameterName`((self, ...) | Get the name of a global parameter. |
    | `getNonbondedMethod`(...) | Get the method used for handling long range nonbonded interactions. |
    | `getNumAcceptors`((self) -> int) | Get the number of acceptors for which force field parameters have been defined. |
    | `getNumDonors`((self) -> int) | Get the number of donors for which force field parameters have been defined. |
    | `getNumExclusions`((self) -> int) | Get the number of donor-acceptor pairs whose interactions should be excluded. |
    | `getNumFunctions`((self) -> int) | Get the number of tabulated functions that have been defined. |
    | `getNumGlobalParameters`((self) -> int) | Get the number of global parameters that the interaction depends on. |
    | `getNumPerAcceptorParameters`((self) -> int) | Get the number of per-acceptor parameters that the interaction depends on. |
    | `getNumPerDonorParameters`((self) -> int) | Get the number of per-donor parameters that the interaction depends on. |
    | `getNumTabulatedFunctions`((self) -> int) | Get the number of tabulated functions that have been defined. |
    | `getPerAcceptorParameterName`((self, ...) | Get the name of a per-acceptor parameter. |
    | `getPerDonorParameterName`((self, ...) | Get the name of a per-donor parameter. |
    | `getTabulatedFunction`((self, ...) | getTabulatedFunction(self, index) -> TabulatedFunction |
    | `getTabulatedFunctionName`((self, ...) | Get the name of a tabulated function that may appear in the energy expression. |
    | `setAcceptorParameters`(self, index, a1, a2, ...) | setAcceptorParameters(self, index, a1, a2, a3) |
    | `setCutoffDistance`(self, distance) | Set the cutoff distance (in nm) being used. |
    | `setDonorParameters`(self, index, d1, d2, d3, ...) | setDonorParameters(self, index, d1, d2, d3) |
    | `setEnergyFunction`(self, energy) | Set the algebraic expression that gives the interaction energy between a donor and an acceptor |
    | `setExclusionParticles`(self, index, donor, ...) | Get the donor and acceptor in a pair whose interaction should be excluded. |
    | `setForceGroup`(self, group) | Set the force group this Force belongs to. |
    | `setFunctionParameters`(self, index, name, ...) | Set the parameters for a tabulated function that may appear in the energy expression. |
    | `setGlobalParameterDefaultValue`(self, index, ...) | Set the default value of a global parameter. |
    | `setGlobalParameterName`(self, index, name) | Set the name of a global parameter. |
    | `setNonbondedMethod`(self, method) | Set the method used for handling long range nonbonded interactions. |
    | `setPerAcceptorParameterName`(self, index, name) | Set the name of a per-acceptor parameter. |
    | `setPerDonorParameterName`(self, index, name) | Set the name of a per-donor parameter. |
    | `updateParametersInContext`(self, context) | Update the per-donor and per-acceptor parameters in a Context to match those stored in this Force object. |
    | `usesPeriodicBoundaryConditions`((self) -> bool) | Returns whether or not this force makes use of periodic boundary conditions. |

    Attributes

    |  |  |
    | --- | --- |
    | `CutoffNonPeriodic` |  |
    | `CutoffPeriodic` |  |
    | `NoCutoff` |  |

    `getNumDonors`(*self*) → int¶
    :   Get the number of donors for which force field parameters have been defined.

    `getNumAcceptors`(*self*) → int¶
    :   Get the number of acceptors for which force field parameters have been defined.

    `getNumExclusions`(*self*) → int¶
    :   Get the number of donor-acceptor pairs whose interactions should be excluded.

    `getNumPerDonorParameters`(*self*) → int¶
    :   Get the number of per-donor parameters that the interaction depends on.

    `getNumPerAcceptorParameters`(*self*) → int¶
    :   Get the number of per-acceptor parameters that the interaction depends on.

    `getNumGlobalParameters`(*self*) → int¶
    :   Get the number of global parameters that the interaction depends on.

    `getNumTabulatedFunctions`(*self*) → int¶
    :   Get the number of tabulated functions that have been defined.

    `getNumFunctions`(*self*) → int¶
    :   Get the number of tabulated functions that have been defined.

    `getEnergyFunction`(*self*) → std::string const &¶
    :   Get the algebraic expression that gives the interaction energy between a donor and an acceptor

    `setEnergyFunction`(*self*, *energy*)¶
    :   Set the algebraic expression that gives the interaction energy between a donor and an acceptor

    `getNonbondedMethod`(*self*) → OpenMM::CustomHbondForce::NonbondedMethod¶
    :   Get the method used for handling long range nonbonded interactions.

    `setNonbondedMethod`(*self*, *method*)¶
    :   Set the method used for handling long range nonbonded interactions.

    `getCutoffDistance`(*self*) → double¶
    :   Get the cutoff distance (in nm) being used. All interactions for which the distance between d1 and a1 is greater than the cutoff will be ignored. If the NonbondedMethod in use is NoCutoff, this value will have no effect.

        |  |  |
        | --- | --- |
        | Returns: | the cutoff distance, measured in nm |
        | Return type: | double |

    `setCutoffDistance`(*self*, *distance*)¶
    :   Set the cutoff distance (in nm) being used. All interactions for which the distance between d1 and a1 is greater than the cutoff will be ignored. If the NonbondedMethod in use is NoCutoff, this value will have no effect.

        |  |  |
        | --- | --- |
        | Parameters: | **distance** (*double*) – the cutoff distance, measured in nm |

    `addPerDonorParameter`(*self*, *name*) → int¶
    :   Add a new per-donor parameter that the interaction may depend on.

        |  |  |
        | --- | --- |
        | Parameters: | **name** (*string*) – the name of the parameter |
        | Returns: | the index of the parameter that was added |
        | Return type: | int |

    `getPerDonorParameterName`(*self*, *index*) → std::string const &¶
    :   Get the name of a per-donor parameter.

        |  |  |
        | --- | --- |
        | Parameters: | **index** (*int*) – the index of the parameter for which to get the name |
        | Returns: | the parameter name |
        | Return type: | string |

    `setPerDonorParameterName`(*self*, *index*, *name*)¶
    :   Set the name of a per-donor parameter.

        |  |  |
        | --- | --- |
        | Parameters: | - **index** (*int*) – the index of the parameter for which to set the name - **name** (*string*) – the name of the parameter |

    `addPerAcceptorParameter`(*self*, *name*) → int¶
    :   Add a new per-acceptor parameter that the interaction may depend on.

        |  |  |
        | --- | --- |
        | Parameters: | **name** (*string*) – the name of the parameter |
        | Returns: | the index of the parameter that was added |
        | Return type: | int |

    `getPerAcceptorParameterName`(*self*, *index*) → std::string const &¶
    :   Get the name of a per-acceptor parameter.

        |  |  |
        | --- | --- |
        | Parameters: | **index** (*int*) – the index of the parameter for which to get the name |
        | Returns: | the parameter name |
        | Return type: | string |

    `setPerAcceptorParameterName`(*self*, *index*, *name*)¶
    :   Set the name of a per-acceptor parameter.

        |  |  |
        | --- | --- |
        | Parameters: | - **index** (*int*) – the index of the parameter for which to set the name - **name** (*string*) – the name of the parameter |

    `addGlobalParameter`(*self*, *name*, *defaultValue*) → int¶
    :   Add a new global parameter that the interaction may depend on.

        |  |  |
        | --- | --- |
        | Parameters: | - **name** (*string*) – the name of the parameter - **defaultValue** (*double*) – the default value of the parameter |
        | Returns: | the index of the parameter that was added |
        | Return type: | int |

    `getGlobalParameterName`(*self*, *index*) → std::string const &¶
    :   Get the name of a global parameter.

        |  |  |
        | --- | --- |
        | Parameters: | **index** (*int*) – the index of the parameter for which to get the name |
        | Returns: | the parameter name |
        | Return type: | string |

    `setGlobalParameterName`(*self*, *index*, *name*)¶
    :   Set the name of a global parameter.

        |  |  |
        | --- | --- |
        | Parameters: | - **index** (*int*) – the index of the parameter for which to set the name - **name** (*string*) – the name of the parameter |

    `getGlobalParameterDefaultValue`(*self*, *index*) → double¶
    :   Get the default value of a global parameter.

        |  |  |
        | --- | --- |
        | Parameters: | **index** (*int*) – the index of the parameter for which to get the default value |
        | Returns: | the parameter default value |
        | Return type: | double |

    `setGlobalParameterDefaultValue`(*self*, *index*, *defaultValue*)¶
    :   Set the default value of a global parameter.

        |  |  |
        | --- | --- |
        | Parameters: | - **index** (*int*) – the index of the parameter for which to set the default value - **defaultValue** (*double*) – the default value of the parameter |

    `addDonor`(*self*, *d1*, *d2*, *d3*, *parameters*) → int¶
    :   addDonor(self, d1, d2, d3) -> int

        Add a donor group to the force

        |  |  |
        | --- | --- |
        | Parameters: | - **d1** (*int*) – the index of the first particle for this donor group - **d2** (*int*) – the index of the second particle for this donor group. If the group only includes one particle, this must be -1. - **d3** (*int*) – the index of the third particle for this donor group. If the group includes less than three particles, this must be -1. - **parameters** (*vector< double >*) – the list of per-donor parameter values for the new donor |
        | Returns: | the index of the donor that was added |
        | Return type: | int |

    `getDonorParameters`(*self*, *index*)¶
    :   Get the properties of a donor group.

        |  |  |
        | --- | --- |
        | Parameters: | **index** (*int*) – the index of the donor group to get |
        | Returns: | - **d1** (*int*) –   the index of the first particle for this donor group - **d2** (*int*) –   the index of the second particle for this donor group. If the group only includes one particle, this will be -1. - **d3** (*int*) –   the index of the third particle for this donor group. If the group includes less than three particles, this will be -1. - **parameters** (*vector< double >*) –   the list of per-donor parameter values for the donor |

    `setDonorParameters`(*self*, *index*, *d1*, *d2*, *d3*, *parameters*)¶
    :   setDonorParameters(self, index, d1, d2, d3)

        Set the properties of a donor group.

        |  |  |
        | --- | --- |
        | Parameters: | - **index** (*int*) – the index of the donor group to set - **d1** (*int*) – the index of the first particle for this donor group - **d2** (*int*) – the index of the second particle for this donor group. If the group only includes one particle, this must be -1. - **d3** (*int*) – the index of the third particle for this donor group. If the group includes less than three particles, this must be -1. - **parameters** (*vector< double >*) – the list of per-donor parameter values for the donor |

    `addAcceptor`(*self*, *a1*, *a2*, *a3*, *parameters*) → int¶
    :   addAcceptor(self, a1, a2, a3) -> int

        Add an acceptor group to the force

        |  |  |
        | --- | --- |
        | Parameters: | - **a1** (*int*) – the index of the first particle for this acceptor group - **a2** (*int*) – the index of the second particle for this acceptor group. If the group only includes one particle, this must be -1. - **a3** (*int*) – the index of the third particle for this acceptor group. If the group includes less than three particles, this must be -1. - **parameters** (*vector< double >*) – the list of per-acceptor parameter values for the new acceptor |
        | Returns: | the index of the acceptor that was added |
        | Return type: | int |

    `getAcceptorParameters`(*self*, *index*)¶
    :   Get the properties of an acceptor group.

        |  |  |
        | --- | --- |
        | Parameters: | **index** (*int*) – the index of the acceptor group to get |
        | Returns: | - **a1** (*int*) –   the index of the first particle for this acceptor group - **a2** (*int*) –   the index of the second particle for this acceptor group. If the group only includes one particle, this will be -1. - **a3** (*int*) –   the index of the third particle for this acceptor group. If the group includes less than three particles, this will be -1. - **parameters** (*vector< double >*) –   the list of per-acceptor parameter values for the acceptor |

    `setAcceptorParameters`(*self*, *index*, *a1*, *a2*, *a3*, *parameters*)¶
    :   setAcceptorParameters(self, index, a1, a2, a3)

        Set the properties of an acceptor group.

        |  |  |
        | --- | --- |
        | Parameters: | - **index** (*int*) – the index of the acceptor group to set - **a1** (*int*) – the index of the first particle for this acceptor group - **a2** (*int*) – the index of the second particle for this acceptor group. If the group only includes one particle, this must be -1. - **a3** (*int*) – the index of the third particle for this acceptor group. If the group includes less than three particles, this must be -1. - **parameters** (*vector< double >*) – the list of per-acceptor parameter values for the acceptor |

    `addExclusion`(*self*, *donor*, *acceptor*) → int¶
    :   Add a donor-acceptor pair to the list of interactions that should be excluded.

        |  |  |
        | --- | --- |
        | Parameters: | - **donor** (*int*) – the index of the donor to exclude - **acceptor** (*int*) – the index of the acceptor to exclude |
        | Returns: | the index of the exclusion that was added |
        | Return type: | int |

    `getExclusionParticles`(*self*, *index*)¶
    :   Get the donor and acceptor in a pair whose interaction should be excluded.

        |  |  |
        | --- | --- |
        | Parameters: | **index** (*int*) – the index of the exclusion for which to get donor and acceptor indices |
        | Returns: | - **donor** (*int*) –   the index of the donor - **acceptor** (*int*) –   the index of the acceptor |

    `setExclusionParticles`(*self*, *index*, *donor*, *acceptor*)¶
    :   Get the donor and acceptor in a pair whose interaction should be excluded.

        |  |  |
        | --- | --- |
        | Parameters: | - **index** (*int*) – the index of the exclusion for which to get donor and acceptor indices - **donor** (*int*) – the index of the donor - **acceptor** (*int*) – the index of the acceptor |

    `addTabulatedFunction`(*self*, *name*, *function*) → int¶
    :   Add a tabulated function that may appear in the energy expression.

        |  |  |
        | --- | --- |
        | Parameters: | - **name** (*string*) – the name of the function as it appears in expressions - **function** (*TabulatedFunction \**) – a TabulatedFunction object defining the function. The TabulatedFunction should have been created on the heap with the “new” operator. The Force takes over ownership of it, and deletes it when the Force itself is deleted. |
        | Returns: | the index of the function that was added |
        | Return type: | int |

    `getTabulatedFunction`(*self*, *index*) → TabulatedFunction¶
    :   getTabulatedFunction(self, index) -> TabulatedFunction

        Get a reference to a tabulated function that may appear in the energy expression.

        |  |  |
        | --- | --- |
        | Parameters: | **index** (*int*) – the index of the function to get |
        | Returns: | the TabulatedFunction object defining the function |
        | Return type: | TabulatedFunction |

    `getTabulatedFunctionName`(*self*, *index*) → std::string const &¶
    :   Get the name of a tabulated function that may appear in the energy expression.

        |  |  |
        | --- | --- |
        | Parameters: | **index** (*int*) – the index of the function to get |
        | Returns: | the name of the function as it appears in expressions |
        | Return type: | string |

    `addFunction`(*self*, *name*, *values*, *min*, *max*) → int¶
    :   Add a tabulated function that may appear in the energy expression.

    `getFunctionParameters`(*self*, *index*)¶
    :   Get the parameters for a tabulated function that may appear in the energy expression.

    `setFunctionParameters`(*self*, *index*, *name*, *values*, *min*, *max*)¶
    :   Set the parameters for a tabulated function that may appear in the energy expression.

    `updateParametersInContext`(*self*, *context*)¶
    :   Update the per-donor and per-acceptor parameters in a Context to match those stored in this Force object. This method provides an efficient method to update certain parameters in an existing Context without needing to reinitialize it. Simply call setDonorParameters() and setAcceptorParameters() to modify this object’s parameters, then call updateParametersInContext() to copy them over to the Context.

        This method has several limitations. The only information it updates is the values of per-donor and per-acceptor parameters. All other aspects of the Force (the energy function, nonbonded method, cutoff distance, etc.) are unaffected and can only be changed by reinitializing the Context. The set of particles involved in a donor or acceptor cannot be changed, nor can new donors or acceptors be added.

    `usesPeriodicBoundaryConditions`(*self*) → bool¶
    :   Returns whether or not this force makes use of periodic boundary conditions.

        |  |  |
        | --- | --- |
        | Returns: | true if force uses PBC and false otherwise |
        | Return type: | bool |

    `__delattr__`¶
    :   x.\_\_delattr\_\_(‘name’) <==> del x.name

    `__format__`()¶
    :   default object formatter

    `__getattribute__`¶
    :   x.\_\_getattribute\_\_(‘name’) <==> x.name

    `__hash__`¶

    `__reduce__`()¶
    :   helper for pickle

    `__reduce_ex__`()¶
    :   helper for pickle

    `__sizeof__`() → int¶
    :   size of object in memory, in bytes

    `__str__`¶

    `getForceGroup`(*self*) → int¶
    :   Get the force group this Force belongs to.

    `setForceGroup`(*self*, *group*)¶
    :   Set the force group this Force belongs to.

        |  |  |
        | --- | --- |
        | Parameters: | **group** (*int*) – the group index. Legal values are between 0 and 31 (inclusive). |

High performance molecular simulation on GPUs


### Navigation

- Application Layer
- Library Layer
  - Core Objects
  - Forces
  - Integrators
  - Extras

©2015, Stanford University and the Authors.
|
Powered by Sphinx 1.3.1
& Alabaster 0.7.7
|
Page source
